# Chromosome-level genome assembly of a kombucha isolate of *Zygotorulaspora florentina*

**DOI:** 10.1128/mra.00232-26

**Published:** 2026-04-23

**Authors:** Steve A. James, Nerice Ryan, Dave J. Baker, Arjan Narbad

**Affiliations:** 1 Food, Microbiome and Health Programme, Quadram Institute Bioscience 7308, Norwich, United Kingdom; 2 Centre for Microbial Interactions, Quadram Institute Bioscience 7308, Norwich, United Kingdom; 3 School of Biological Sciences, University of East Anglia 6106, Norwich, United Kingdom; University of Strathclyde, Glasgow, United Kingdom

**Keywords:** fungi, fermented foods, kombucha, probiotics, *Zygotorulaspora florentina*, genome sequencing

## Abstract

*Zygotorulaspora florentina* is a fermentative yeast of growing interest to both the brewing and winemaking industries. Here, we report the draft genome sequence of *Z. florentina* NCYC 4520, a kombucha-derived isolate.

## ANNOUNCEMENT

*Zygotorulaspora florentina*, formerly *Zygosaccharomyces florentinus* (1), is an ascomycetous yeast that has been isolated from a variety of sources, including grape must, plant material, fruit flies, and fruit-based soft drinks (2). With its ability to ferment maltose and maltotriose, as well as enhance aroma complexity in co-fermentations with *Saccharomyces cerevisiae*, there is growing interest in its potential use in both brewing and winemaking (3–5). Here, we combined short- and long-read sequencing to assemble the genome sequence of *Z. florentina* NCYC 4520, isolated from a bottle of Norfolk-produced organic kombucha purchased in Norwich, UK. A 100 μL kombucha sample was plated onto Rose Bengal agar and incubated for 2 days at 25°C. Colonies were picked and purified by two additional rounds of re-streaking on Yeast Malt (YM) agar (10 g/L glucose, 3 g/L malt extract, 5 g/L peptone, and 3 g/L yeast extract) and incubated at 25°C. The species was identified by ITS sequencing using primers ITS1 and ITS4 (6, 7) (GenBank accession number PX480735). Sequence identity was 100% to the *Z. florentina* type strain (KY106082), based on a BLASTn query (8) against the NCBI rRNA_typestrains/ITS_RefSeq_Fungi database.

Genomic DNA was isolated using a MasterPure yeast DNA purification kit (Cambio), with additional zymolyase and proteinase K treatment steps, from cells harvested from a 2-day liquid YM culture grown at 25°C. A short-read library was prepared using a Nextera DNA Flex library prep kit (Illumina) with 20-fold diluted reagents and sequenced on an Illumina NextSeq 500, producing 11.6 million paired-end 150 bp reads. A Nanopore library was prepared from the same gDNA prep without fragmentation or size selection, using the SQK-LSK109 1D ligation kit (Oxford Nanopore Technologies, ONT), and sequenced on a MinION instrument (ONT) with a R9.4.1 flow cell (ONT). This produced 336,776 reads with an N_50_ of 15,734 bp. Default parameters were used for all software unless otherwise specified. Base calling was performed using Guppy (ONT; v3.6.0) in high-accuracy mode (model dna_r9.4.1_450bps_hac).

Illumina reads were filtered using fastp v.0.23.2 (9). Nanopore reads were filtered using NanoFilt v.2.3.0 (10), selecting reads with quality ≥10 and length ≥5,000 bp. Read quality was verified with FastQC v.0.12.1 (11). After filtration, 161,449 Nanopore reads were retained (mean length = 14,386 bp). *De novo* assembly of Nanopore reads was performed using Flye v2.9.1 (12), using long reads in “ONT regular reads, pre-Guppy5” mode. The assembly was polished with the Illumina data using three rounds of alignment with BWA-MEM v.0.7.17.1 (13) and correction with Pilon v.1.24 (14). The final assembly consists of 15 contigs, including 8 chromosome-sized contigs, 5 with a 13 bp telomeric repeat sequence (5′-TGTAGGGGTGTGG-3′)_n_ (15), identified using Tandem Repeats Finder v.4.09 (16), at one end. The total length is 11,137,782 bp, with an N_50_ of 1,404,365 bp, and a G + C content of 41.03%. Genome completeness was estimated at 99.6% using BUSCO v.5.5.0 (17) with the saccharomycete_odb10 data set. Augustus v.3.2.3 (18) predicted 5,065 protein-coding genes using the *Saccharomyces cerevisiae* training data set, and 194 tRNA genes were detected using tRNAscan-SE 2.0 (19).

## Data Availability

This whole-genome shotgun project has been deposited in DDJB/ENA/GenBank (BioProject number PRJNA1333318, assembly accession number JBVFKW000000000). The version described in this paper is version 1. The raw reads are available in the NCBI Sequence Read Archive (SRA) under accession numbers SRX30640826 (ONT) and SRX30640825 (Illumina). The predicted protein and tRNA annotation data sets have been deposited in Zenodo (https://doi.org/10.5281/zenodo.18151368).
